# Development of an Analytic Convection Model for a Heated Multi-Hole Probe for Aircraft Applications

**DOI:** 10.3390/s21186218

**Published:** 2021-09-16

**Authors:** Pablo Nieto Muro, Florian M. Heckmeier, Sean Jenkins, Christian Breitsamter

**Affiliations:** 1 Chair of Aerodynamics and Fluid Mechanics, TUM Department of Mechanical Engineering, Technical University of Munich, Boltzmannstr. 15, D-85748 Garching, Germany; pablonieto.tum@gmail.com (P.N.M.); christian.breitsamter@aer.mw.tum.de (C.B.); 2 Vectoflow GmbH, Friedrichshafener Str. 1, D-82205 Gilching, Germany

**Keywords:** ice accretion, icing, multi-hole probe, anti-icing, heat transfer, convection, additive manufacturing, standard atmosphere, dimensional analysis

## Abstract

Ice accretion or icing is a well-known phenomenon that entails a risk for the correct functioning of an aircraft. One of the areas more vulnerable to icing is the air data measuring system. This paper studies the icing protection offered by a heating system installed inside a multi-hole probe. The problem is initially solved analytically, creating a tool that can be used in order to predict the heating performance depending on the flying conditions. Later, the performance of the real system is investigated with a heated five-hole probe prototype in a wind tunnel experiment. The measured results are compared with the predictions made by the analytical model. Last, the icing protection provided by the system is estimated with respect to flying altitude and speed. As a result, a prediction tool that can be used in order to make quick icing risk predictions for straight cylindrical probes is delivered. Furthermore, the study provides some understanding about how parameters like altitude and air speed affect the occurrence of ice accretion.

## 1. Introduction and Motivation

In flight control, the onboard measurement of air speed and angle of attack is necessary in order to estimate the drag and lift forces that determine the movement of an aircraft [1,2]. In the case of aircraft likely to operate under harsh conditions, such as Unmanned Aerial Vehicles (UAV), the measurement of these flight control parameters is usually performed by multi-hole probes [3,4]. The reason is their structural simplicity and robustness in comparison with other measurement techniques.

Multi-hole probes are pressure-based velocity measurement systems. They are often used in cases where the flow direction is unknown or presents large variation [5]. Multi-hole probes normally consist of a slender body containing a series of tubes called pressure channels. The channels extend parallel to each other until the head of the probe, where they are open to the environment. These openings are called the pressure ports. Given that pressure varies with altitude, probes used in aircraft applications are equipped with an additional series of reading ports, called the static ring, where static pressure is measured. The probes are calibrated before their usage in flight conditions and can resolve flow angles up to $\pm 60^{{}^{\circ}}$ with high precision [6].

Atmosphere conditions related to altitude can be determined by the U.S. Standard Atmosphere of 1976, which is an idealized, steady-state representation of the atmosphere that provides valid relations between these parameters [7]. From a pre-study, the UAV typical service ceiling is determined to be between 10,000 and 30,000 ft. According to U.S. Standard Atmosphere data [7], these altitudes correspond to typical temperatures of −4.8 ${\;}^{{}^{\circ}}$C and −44.4 ${}^{{}^{\circ}}$C. For these conditions, ice accretion is likely to take place.

Ice accretion, or icing, is a well-known phenomenon in aviation since it can have negative consequences for many exposed aircraft systems [8]. At temperatures below freezing, water may still remain liquid, as supercooled droplets. This state is unstable and these droplets may freeze abruptly when coming in contact with a solid surface [9]. The obstruction of one or more of the pressure ports in a probe can lead to the corruption of the system readings [10]. The normally chosen Icing Protection System (IPS) used to protect this type of system is thermo-electric protection [9]. This consists of heating the probe surface by circulating an electric current through a resistive element. The IPS can be classified into de-icing or anti-icing depending on whether they allow the ice to build up or not. If the resulting surface temperature is high enough to evaporate the impinging water, the system is said to be evaporative and corresponds to the de-icing category. If the temperature is only high enough to prevent the solidification, letting the liquid water flow over the surface driven by the flow aerodynamic forces, the system is called wet runback and belongs to the anti-icing category. Anti-icing systems require less heating power and are therefore lighter, but they cannot remove formed ice on the probe surface as de-icing systems can.

**Figure 1:**
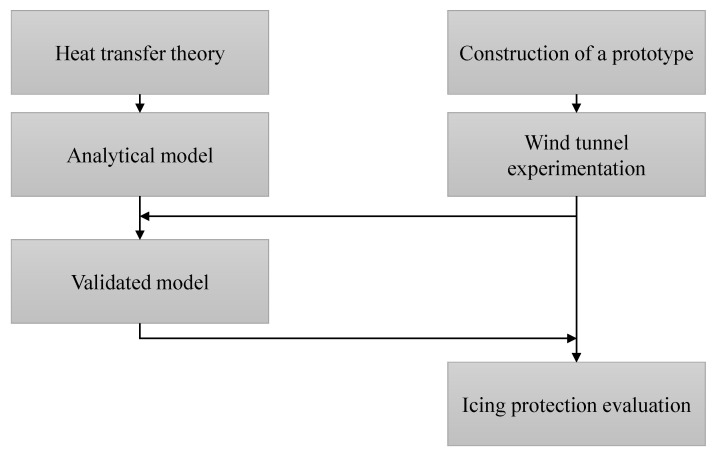
Organisation of the performed study.

This paper characterizes the effect of adding an anti-icing system to a multi-hole probe in collaboration with the probe manufacturer Vectoflow GmbH. In the first part of the paper, the heat transfer problem between the heating system and the probe environment is studied in order to present an analytical model that makes system performance predictions depending on the aircraft flight conditions. Thereafter, the system is tested in the subsonic *Wind Tunnel B* (W/T-B) of the Chair of Aerodynamics and Fluid Mechanics of the Technical University of Munich (TUM-AER) in order to measure its real performance. For that purpose, a heated probe prototype is developed and instrumented in order to perform the necessary measurements during the experimentation. In the last part, the analytical model validity is discussed, the anti-icing protection provided is estimated and an outlook is given. The study structure is represented in Figure 1.

## 2. Theory and Analytical Model

In this section, the underlying analytic convection problem is mathematically explained and characterized. As mentioned, a heating system should be included in a multi-hole probe in order to protect it from icing. In order to keep ice sensitive areas protected, electrical anti-icing systems need to provide enough heat to the surfaces so that their temperature is maintained sufficiently over the freezing point [11]. In order to make estimations for this temperature with the heating system activated, the related heat transfer theory and the design of a prediction model are presented in the following.

### 2.1. Heat Convection Theory

The thermal problem that presents the heat exchange from an electrically heated surface to an airflow is a convection problem. The term convection is used to describe energy transfer between a surface and a fluid moving over this surface [12]. The convection heat flux $q^{\prime \prime }$ is defined as

(1) $$q^{\prime \prime }=\frac{dq}{dA}=h\;\left(T_{S}-T_{\infty }\right),$$

where $T_{\infty }$ is the free stream temperature, $T_{S}$ the surface temperature and *h* the convection heat transfer coefficient. The total heat transfer rate across an isothermal surface of area $A_{s}$ can be obtained by integrating Equation (1) as

(2) $$q=\left(T_{S}-T_{\infty }\right)\;\int_{A_{S}}h\;dA_{S}.$$

With the definition of an average convection heat transfer coefficient $\bar{h}$, Equation (2) can be rewritten as:

(3) $$q=\bar{h}\;A_{S}\;\left(T_{S}-T_{\infty }\right).$$

Complexity regarding convection problems normally lies in determining the values of *h* or $\bar{h}$, since these depend on three main groups: fluid properties, surface geometry and boundary layer regime [12]. As a consequence, heat convection problems can be solved analytically only for a limited number of elementary cases. For problems involving turbulent flow or separation—for example around cylinders, spheres or other curved bodies—the direct measuring of the heat transfer coefficient is still the main approach employed [13]. The experimental results are presented in the form of empirical relations that do not rely on theory but on measurements. These expressions are obtained using the method of dimensional analysis.

Dimensional analysis provides a method for computing sets of dimensionless parameters that describe a problem defined by a certain set of variables [14]. For its application, the variables affecting the phenomenon must be known beforehand [13]. Experience tells us that the convection coefficient *h* is a function of the velocity *V*, viscosity μ, density ρ and thermal conductivity *k* of the fluid and the characteristic dimension *d*:

(4) h=f(V,μ,ρ,k,d).

This expression may be rewritten after applying dimensional analysis in the form:

(5) $$\frac{hd}{k}=C\;{\left(\frac{\rho Vd}{\mu }\right)}^{a}\;{\left(\frac{\mu c_{p}}{k}\right)}^{b},$$

where *C*, *a* and *b* are undefined coefficients. The three dimensionless terms in Equation (5) are the Nusselt number $Nu=\frac{hd}{k}$, the Reynolds number $Re=\frac{\rho Vd}{\mu }$ and the Prandtl number $Pr=\frac{\mu c_{p}}{k}$. Therefore, experimental testing results are usually generalized by establishing the relationship between the Nusselt and the Reynolds and the Prandtl numbers [15]:

(6) $$Nu=f\left(Re,Pr\right)=C\cdot Re^{a}\cdot Pr^{b}.$$

Each of the three dimensionless parameters has a physical significance. The Reynolds number represents the ratio between inertial and viscous forces. Its value can be used in order to predict the transition of the boundary layer regime [12]. The critical Reynolds number $Re_{c}$ at which the flow becomes turbulent depends on the geometric configuration. For example, for tube flows, it lies around $Re_{c}\approx 2300$, and for flows over a flat plate it occurs at $Re_{c}\approx 5\times 10^{5}$ [13]. The Nusselt number gives the ratio of the convective to conductive heat transfer across the fluid boundary. It provides a measure of the convection heat transfer occurring at the surface [13]. In the presence of turbulence, greater flow velocities in the vicinity of the solid surface promote large temperature gradients on the surface [12]. This translates into higher Nu values and consequently also to an increase of the *h* values. Hence, it can be concluded that the turbulent regime increases the convection heat exchange between surface and fluid.

The Prandtl number correlates three fluid properties and thus is a property of the fluid itself [13]. For air at 1 atm and 25 ${}^{{}^{\circ}}$C, its value is Pr=0.707 and does not vary significantly at the conditions investigated within this work.

Furthermore, in order to make surface temperature predictions for the heated probe, it is necessary to elaborate a model for the specific geometry of the probe as discussed in Equation (6). In the following section, existing simple models are used for the derivation of the new probe model. The simple models have been derived experimentally for similar geometries and can be found in the literature.

### 2.2. Heat Convection Model for the Heated Probe

**Figure 2:**
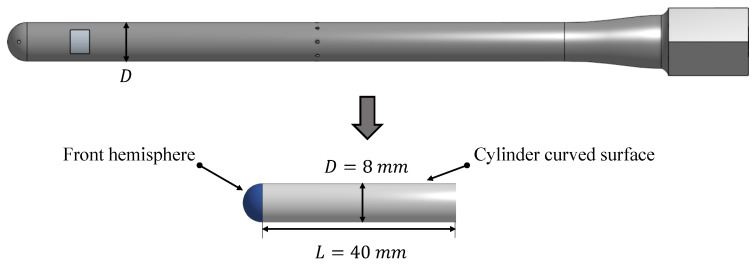
Probe geometry transformation for the definition of the analytical model.

After the presentation of the general introduction to heat convection theory in the previous section, in this section, the heat convection model for a heated pressure probe is elaborated. The underlying problem consists of a straight probe under an axial inflow conditions. The probe has a cylindrical body and a hemispherical head shape. The airflow is assumed to be steady and incompressible. The pressure holes are neglected and a solid probe without pressure tubings is assumed in the following. The probe body is represented by a cylinder directly connected to the hemispherical head. Given that the heater is located at the front part of the probe, it is expected that the entire length of the probe will not be relevant for the formulation of the convection problem. Hence, heat is assumed to be dissipated mostly at the front part. For that reason, the cylinder length is defined as *L*, which represents the length of the probe that is effectively heated. Figure 2 shows the geometrical assumptions made in order to enable the definition of an analytical model.

The total amount of dissipated heat at the transformed probe $q_{total}$ is obtained by summing up the dissipated heat through the front hemisphere $q_{hem}$ and the dissipated heat through the cylinder $q_{cyl}$:

(7) $$q_{total}=q_{hem}+q_{cyl}.$$

Hence, in order to describe the full convection problem, it is necessary to account for two different convection models: one for the front hemisphere and another for the cylinder. The two corresponding models and the resulting analytical model are presented in the following subsections.

#### 2.2.1. The Front Hemisphere

**Figure 3:**
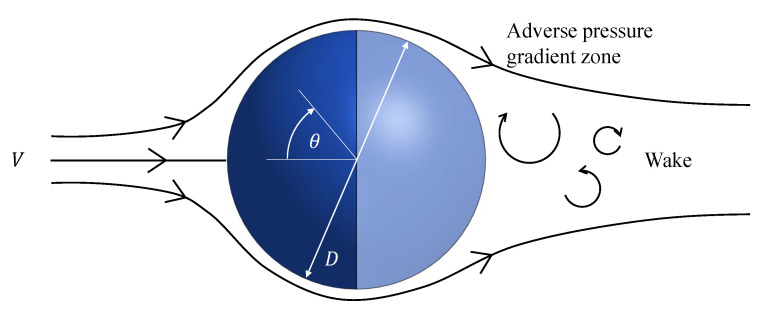
Streamlines around an sphere under a uniform flow.

The first presented model corresponds to the probe head represented by a front hemisphere of the probe diameter *D*. While the flow is well attached to the surface for the front half of the sphere, the adverse pressure gradient at the back half leads to separation and the formation of a wake behind the sphere. These differences cause the proportion of heat dissipated from the front and back halves to be different [16]. A schematic representation of this phenomena is shown in Figure 3.

Eastop and Smith [17] present a heat convection model that differentiates between the front and back halves. This model is also discussed in Will et al. [18]. The model consists of the calculation of the average Nusselt number over a sphere. The estimation is performed by the sum of two terms:

(8) $${\bar{Nu}}_{D}=0.42\;Re_{D}^{0.50}+0.0035\;Re_{D}^{0.92}\;\mathrm{for}\;3.0\times 10^{3}<Re_{D}<1.0\times 10^{5}.$$

The first term accounts for the contribution of the front hemisphere and the second for the back hemisphere. For the studied case, it is desired to obtain a new equation from Equation (8) to estimate $\bar{Nu_{D}}$ exclusively over the front hemisphere. Since the estimated value is an average, it would be sufficient to substitute the term corresponding to the back hemisphere by an additional front hemisphere term. This way, the mean value over the entire sphere corresponds to the mean over the front hemisphere. In Appendix A, additional information and an explanation concerning this assumption is discussed. The final expression gives:

(9) $$\begin{array}{cc} {\bar{Nu}}_{D}= & 0.84\;Re_{D}^{0.50}\;\mathrm{for}\;3.0\times 10^{3}<Re_{D}<1.0\times 10^{5}. \end{array}$$

#### 2.2.2. The Curved Surface of a Cylinder in Axial Flow

**Figure 4:**
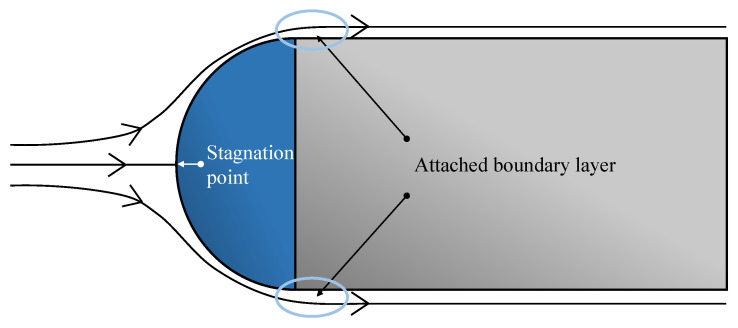
Stagnation point position and expected flow streamlines for the considered geometry under an axial flow.

The second model represents the cylindrical probe body. The case presented by the considered geometry is not exactly equivalent to a cylinder under an axial flow. The flow around the probe geometry is expected to go around the probe head and attach to the cylinder surface without any or small flow separation at the transition between the hemisphere and the cylindrical part. A representation of the expected flow streamlines is shown in Figure 4.

**Figure 5:**
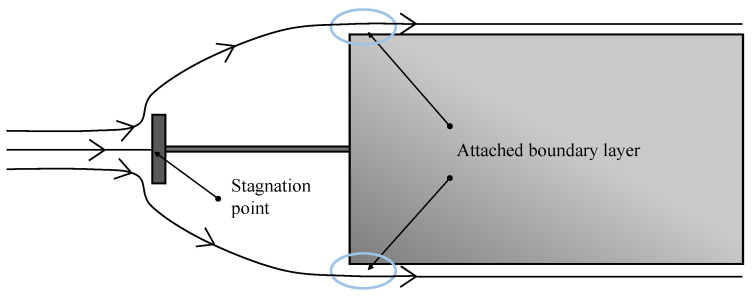
Stagnation point position and flow streamlines for the disc and cylinder under an axial flow [19].

Wiberg et al. [19] introduce a series of experiments for a cylinder of diameter *D* in axial flow. One of the layouts consists of a cylinder with a circular disc located upstream in order to mimic a smooth flow at the cylinder ends. The circular disc has the diameter $D_{disc}=1/3\;D$ and is located a distance *D* upstream from the front of the cylinder under study. The results show that the disc upstream from the cylinder causes the flow streamlines to diverge from the cylinder axis before reaching the cylinder surface. As a consequence, a better attachment of the flow to the leading edge of the cylinder curved surface is obtained. This reduces the separation effect at the junction between the front face and curved faces of the cylinder and it becomes negligible [19]. The flow behavior for this case is depicted in Figure 5.This provides a scenario more similar to the one expected for the considered geometry that is presented in Figure 4. For this adapted configuration, Wiberg et al. elaborate an expression to characterize the heat convection over the cylinder curved surface [19]:

(10) $${\bar{Nu}}_{D}=0.058\;Re_{D}^{0.75}\;\mathrm{for}\;1.77\times 10^{5}<Re_{D}<6.09\times 10^{5}.$$

#### 2.2.3. Resulting Analytical Model

**Figure 6:**
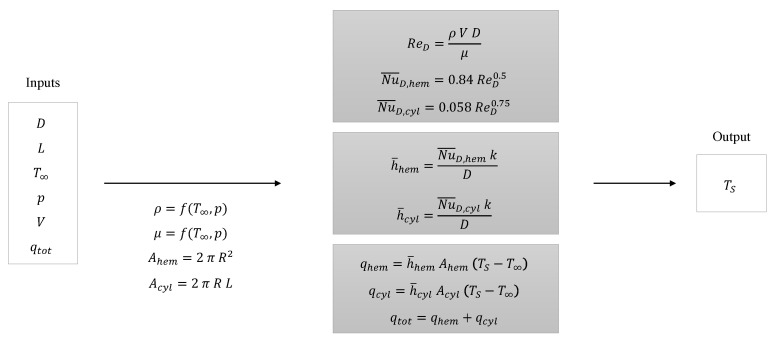
Inputs, computation formulas and output of the analytical model.

With the determination of the two convection models for the two separated geometries, both models can be combined in order to define the resulting analytical model for the probe geometry. The model is based on fundamental convection theory, presented in Section 2.1, and defines a methodology for making $T_{S}$ predictions at the surface of the considered probe geometry represented in Figure 2. As specified in Equation (7), the global model is defined as the combination of two models. These two models are defined by the expressions in Equations (9) and (10). Each of them is used to determine the convected heat through each of the respective geometries using the corresponding terms for the Reynolds and Nusselt numbers in Equations (5) and (6). After adequately combining all the mentioned expressions, the result is a model that takes *D*, *L*, $T_{\infty }$, *p*, *V* and $q_{total}$ as inputs and returns $T_{S}$ as a single output. The thermophysical properties of air are obtained using the software “CoolProp” which is based on Helmholtz energy calculations after inserting the temperature and the pressure for the fluid [20]. The reference temperature used in all Reynolds and Nusselt number estimations is $T_{\infty }$. The computation proceeding is summarized in Figure 6.

## 3. Experimental Setup and Probe Assembly

In order to validate the heat convection model of the previous section, a heated probe prototype is manufactured, instrumented and tested in a wind tunnel. The agreement between the model predictions and the real performance of the system is evaluated by comparing the analytical model output to the acquired experimental data.

Like the rest of the probes manufactured by Vectoflow GmbH, the heated probe prototype is manufactured using the Powder Bed Fusion (PBF) method, being a good example of the direct tooling phase that Additive Manufacturing (AM) has experienced in the last years [21]. This phase corresponds to the application of AM in the production of finished parts. This production technique offers a higher degree of customization and the realization of more complex geometries in comparison with conventional means. Boerner and Niehuis [22] and Heckmeier et al. [23] make use of additive manufacturing advantages by employing Vectoflow probes on their studies.

**Figure 7:**
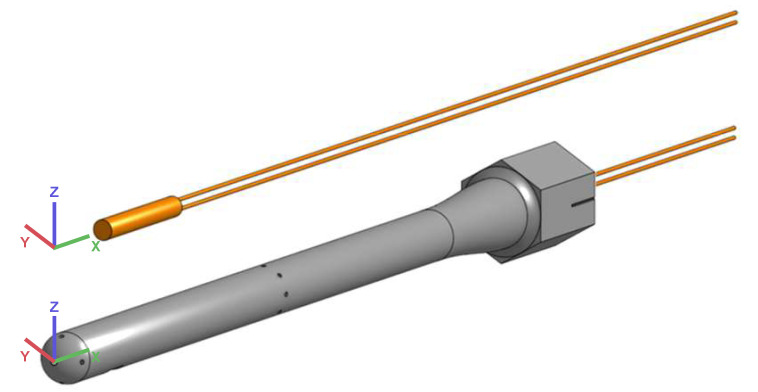
CAD model for the five-hole probe and the heating element.

The prototype consists of a straight five-hole probe with a static ring on its shaft and an axial cavity from the back of the probe with a heating element. The insertion of the heater inside the probe is represented in Figure 7. With minimisation of weight as one of the main design goals, the probe diameter is set to a feasible minimum of D=8 mm and the probe length to L=153 mm.

**Figure 8:**
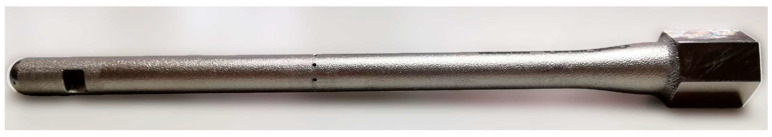
Additive manufactured probe after printing and the side opening to the axial heater cavity.

A 4 mm long cavity connecting the probe surface to the the heater axial cavity is added to the design in order to facilitate the bonding of the heater by offering a way for adhesive introduction during the heater mounting process. This cavity can be observed on the printed part in Figure 8. Additionally, the pressure channels are blocked with wax at the back of the probe in order to avoid the flow of air though them during experimentation.

The heating system performance is evaluated measuring temperature on the probe surface under a series of different heater power intensities and airflow conditions. The experiments with the heated five-hole probe are conducted in the W/T-B of TUM-AER. The low-speed wind tunnel, which is of Göttingen type (closed-loop), has a cross section of h·b=1.20 m ×1.55 m. Turbulence intensity lies below Tu=1%. The incoming free stream velocity *V* is monitored with a standard Prandtl probe installed at the nozzle exit, acquiring the dynamic pressure $p_{dyn}$. Furthermore, a temperature probe (PT100) is installed to acquire flow temperature data $T_{\infty }$. Hence, together with the output of the Prandtl and the temperature probes, the atmospheric pressure signal $p_{s}$ are monitored. The power supplied to the probe heating system is controlled by regulating an external voltage source. The test configurations are depicted in Table 1. The first four configurations have an identical flow velocity *V* while the heater power *q* is increased. In the last two configurations, the heater power is maintained and the airflow speed is stepwise increased.

The temperatures on the probe surface are read by six type K thermocouples [24] mounted at different positions along the length of the probe. The axial coordinate values for each of the measurement points are given in Table 2. The probe tip is defined as the origin (z=0 mm). Therefore, the axial coordinate *z* is also referred to as the distance from the probe tip.

**Figure 9:**
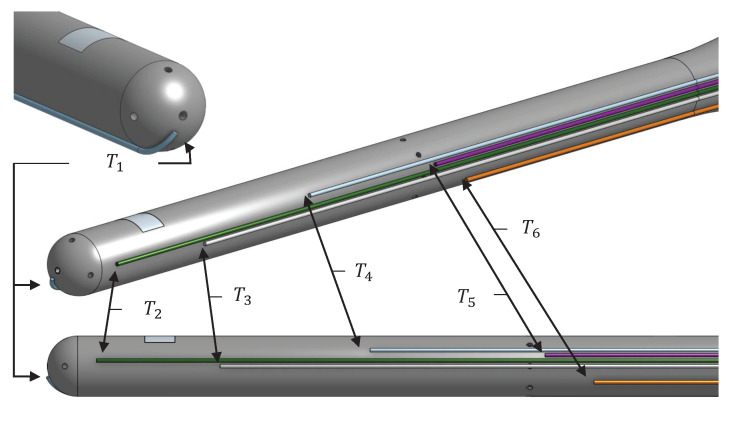
Thermocouples positioning on the probe surface.

The temperature measurement $T_{1}$ is located on the probe head. Then, $T_{2}$, $T_{3}$ and $T_{4}$ are located between the probe head and static ring. Last, $T_{5}$ and $T_{6}$ are located after the static ring, with $T_{5}$ located very close to it. A better understanding of the exact position of the temperature measurement locations is represented in Figure 9. The measuring points are not located over a common axial plane over the surface, since temperatures are expected to show independence with the azimuthal coordinate θ due to axial symmetry.

**Figure 10:**
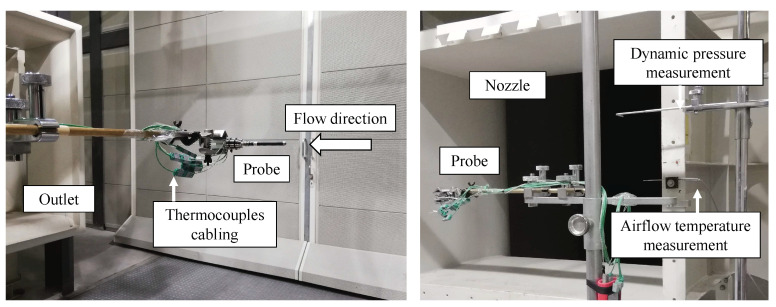
Position of the probe in the wind tunnel test area and relevant setup elements.

During the test, the probe is positioned in the wind tunnel test area aligned with the airflow. Figure 10 shows the final setup. The probe is located centered near the wind tunnel nozzle.

For each test configuration, the wind tunnel is turned on until the desired air speed is reached according to the read dynamic pressure. After reaching thermal equilibrium, the airflow temperature as well as the read temperatures by the thermocouples on the prototype surface are acquired for each configuration.

## 4. Results

In this section, the acquired temperature test results are presented first. Then they are compared with predictions made by the developed analytical heat convection model in order to evaluate the agreement with the real system behavior. Finally, the expected system behavior under real application conditions is estimated. The result of this last step is the generation of icing prediction graphs with respect to flight altitude and speed.

### 4.1. Temperature Measurements

The final test results are presented in Table 3. According to an uncertainty evaluation of the measurement data, all measurements show a deviation lower than $\pm 1^{{}^{\circ}}$C with a 95% confidence level.

**Figure 11:**
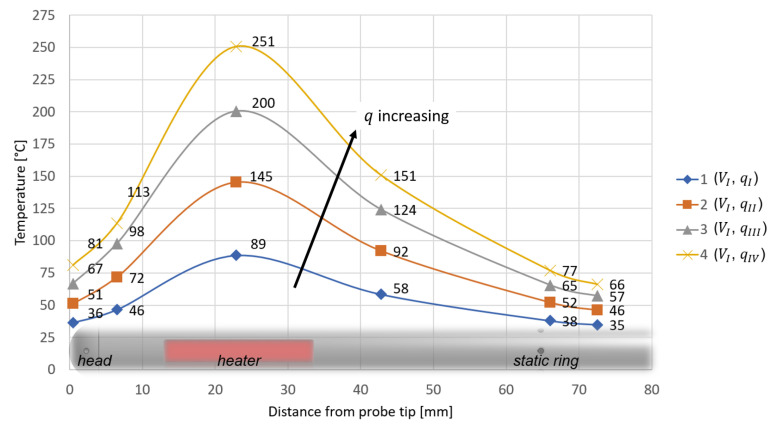
Measured temperature profiles for the test configurations 1, 2, 3 and 4.

**Figure 12:**
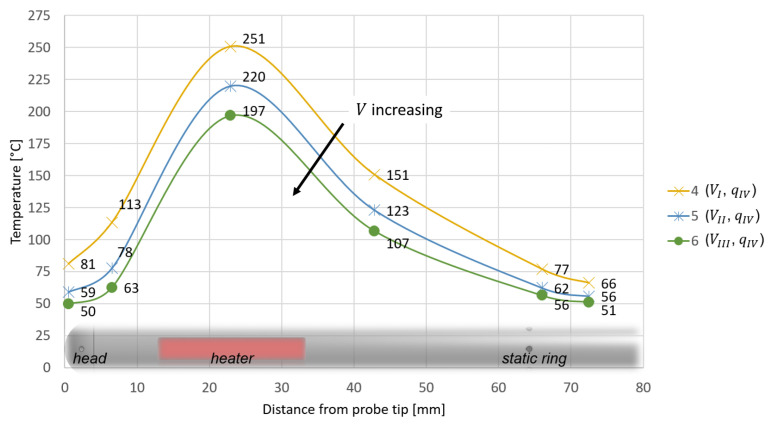
Measured temperature profiles for the test configurations 4, 5 and 6.

The test data are depicted in Figure 11 and Figure 12 with data points, while in addition spline curve fits are added. The resulting temperature profiles are represented with respect to the axial coordinate *z*. For all cases, temperature increases from the probe head to the heater, reaching a maximum, and then decreases as the distance from the probe tip is further increased. Figure 11 shows how temperatures increase as *q* increases, while in Figure 12 temperature trends decrease as *V* is increased.

### 4.2. Comparison to the Analytical Model

**Figure 13:**
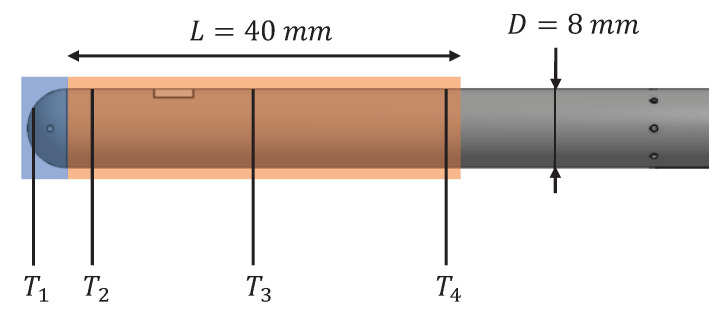
Considered probe length by the analytical model and neighboring measurement points.

The test results are used in order to evaluate the validity of the predictions made by the analytical model. The comparison between the analytical model output and the experimental test results is done by defining a representative temperature $T_{test}$ that approximates the profile mean temperature over the probe length considered by the analytical model. This length is defined as L=5D=40 mm for all test configurations. This length was chosen due to the positions of measurement points and represents the area most influenced by the heating element. Figure 13 represents this length over the probe geometry and the position of the available test reading points in order to determine the most adequate way to define $T_{test}$.

**Figure 14:**
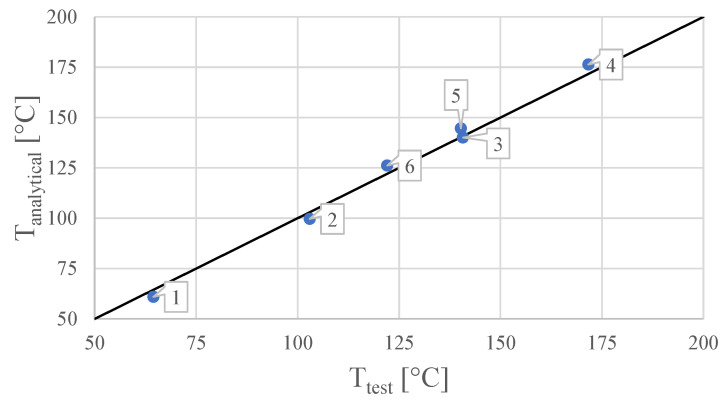
Comparison between the analytical model output $T_{analytical}$ and the corresponding experimental test temperature reading $T_{test}$. The data labels indicate the test configuration, respectively.

Since $T_{2}$, $T_{3}$ and $T_{4}$ present an acceptably even distribution over the analytical model length, the value $T_{test}$ is defined as the mean temperature averaged with these three values. The comparison between $T_{analytical}$ and $T_{test}$ is given in Table 4. The formulas for the calculation of the error ΔT and the relative error δT with respect to $T_{test}$ are given in Equations (11) and (12). The relative error is computed with respect to the temperature difference to set $T_{\infty }$ as the reference. The comparison between $T_{analytical}$ and $T_{test}$ is also represented in Figure 14, where the function $T_{analytical}=T_{test}$ is represented by a line in black in order to show the agreement between the test results and the model.

(11) $$\Delta T=T_{analytical}-T_{test}$$

(12) $$\delta T=\frac{\Delta T}{T_{test}-T_{\infty }}.$$

From the comparison shown in Figure 14, it can be concluded that the analytical model and the test results present a good agreement since all points fall very close to the $T_{analytical}=T_{test}$ line. The results shown in Table 4 show that the relative errors δT vary from −4.4% to +3.0%.

### 4.3. Evaluation of the Heating System Anti-Icing Capability

The air temperature and static pressure inside the TUM-AER wind tunnel test section cannot be adjusted to flight conditions. Hence, in order to translate the test results to typical flight elevation atmospheric conditions, expressions are found in relation to the dimensional analysis problem presented in Section 2.1. For this case, the selected dimensionless number to be conserved between different scenarios is the Reynolds number $Re_{D}$. By preserving Reynolds similarity, the determination of equivalent air speeds is performed as:

(13) $$\begin{array}{c} Re_{D,1}=Re_{D,2} \end{array}$$

(14) $$\begin{array}{c} \frac{V_{1}\cdot D}{\nu_{1}}=\frac{V_{2}\cdot D}{\nu_{2}} \end{array}$$

(15) $$\begin{array}{c} V_{2}=\frac{V_{1}\cdot \nu_{2}}{\nu_{1}}, \end{array}$$

with air properties $\nu_{1}$, $k_{1}$ for scenario 1 and $\nu_{2}$, $k_{2}$ for scenario 2. According to the developed analytical model, $Nu_{D}$ conservation is a direct consequence of the $Re_{D}$ conservation. Furthermore, the dissipated heat at one scenario or another is independent of the air properties; this is also a quantity that remains equal between scenarios. This new consideration results in the following equations which allow the translation of equivalent temperatures between scenarios.

(16) $$\begin{array}{c} q_{1}=q_{2} \end{array}$$

(17) $$\begin{array}{c} h_{1}A\left(T_{1}-T_{\infty ,1}\right)=h_{2}A\left(T_{2}-T_{\infty ,2}\right) \end{array}$$

(18) $$\begin{array}{c} \frac{Nu_{D}k_{1}}{D}A\left(T_{1}-T_{\infty ,1}\right)=\frac{Nu_{D}k_{2}}{D}A\left(T_{2}-T_{\infty ,2}\right) \end{array}$$

(19) $$\begin{array}{c} T_{2}=\frac{k_{1}}{k_{2}}\left(T_{1}-T_{\infty ,1}\right)+T_{\infty ,2}. \end{array}$$

It is desired to study the efficacy of the heating system of the prototype as an anti-icing system. To do this, the temperatures at the probe head and static ring are predicted by using the data measured during the experimentation. The output of the model should be a graph that represents the predicted temperatures depending on the flying conditions, that is, the flight speed and the altitude, which can be related to certain pressure and temperature conditions according to the Standard Atmosphere data. The anti-icing evaluation graph is conceived as the maximum performance that the system can deliver. Therefore, only the data for the system working at maximum capacity is used. This means that from the results presented at the introduction of Section 4, only those from configuration 4, 5 and 6 are considered here. The head and static ring temperatures, $T_{1}$ and $T_{5}$, from these configurations are represented in Table 5 in ${}^{{}^{\circ}}$C.

**Figure 15:**
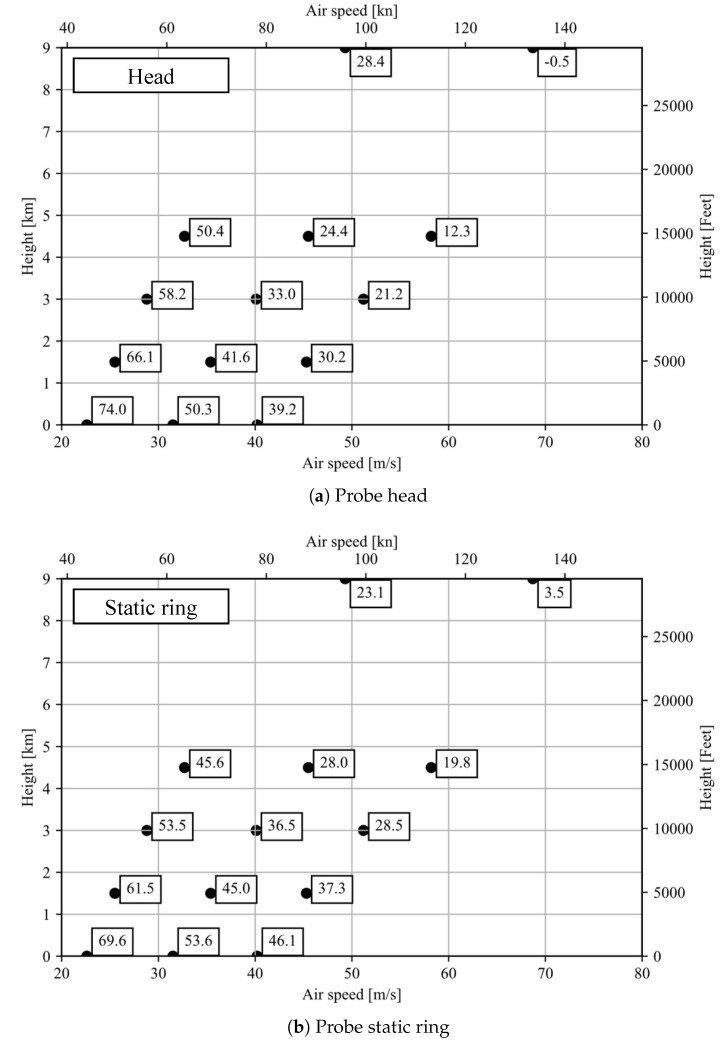
Anti-icing evaluation graph containing test data from configurations 4, 5 and 6 translated to the considered Standard Atmosphere cases in ${}^{{}^{\circ}}$C.

The test data are translated to the Standard Atmosphere cases of 0, 1.5, 3, 4.5 and 9 km elevation using Equations (15) and (19). This is an extrapolation of the data taken in wind tunnel experiments and the results are represented in Figure 15a,b in ${}^{{}^{\circ}}$C.

It can be observed that, as the respective altitude is increased, the translated air speeds increase, displacing the data points to the right. For the 9 km case, the displacement is high enough that the data point matching configuration 6 falls outside the air speed range considered by the graph. For the probe head, this point is located at V=87.8 m/s and has a value of −14.0 ${}^{{}^{\circ}}$C. For the static ring, the point falls at the same speed and its value is −5.6 ${}^{{}^{\circ}}$C.

**Figure 16:**
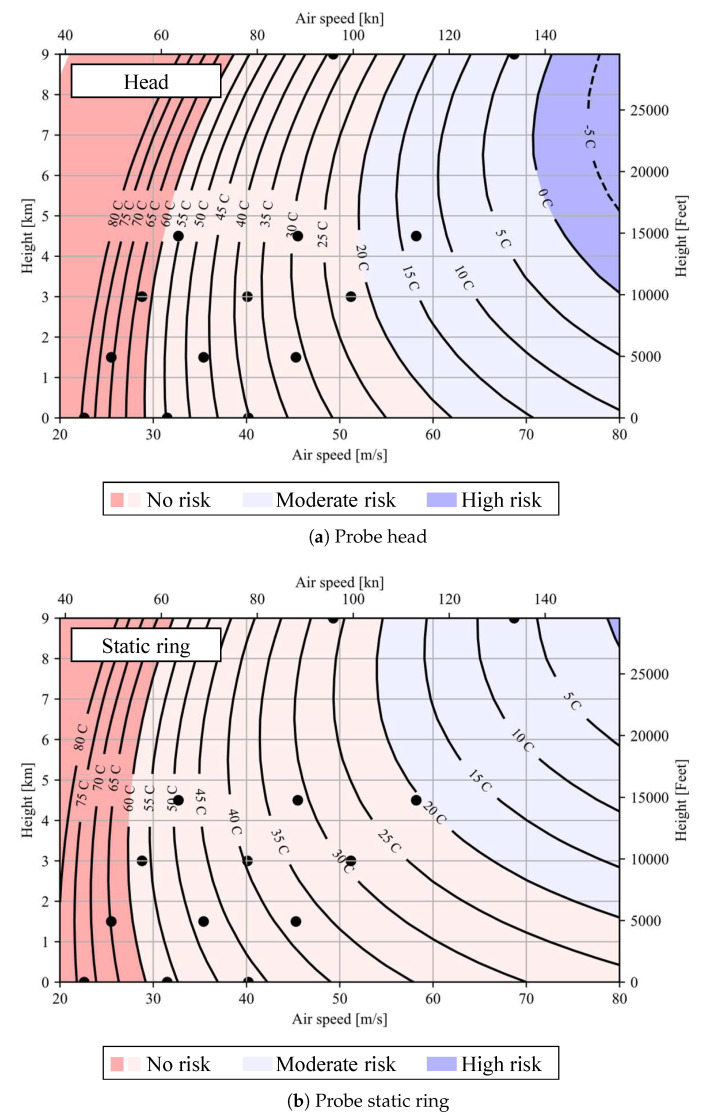
Anti-icing evaluation graph for the probe showing the accordance between the test translated data and the predicted temperature values by the regression model.

Given the good agreement observed between the analytical model predictions and the test data, $T_{analytical}$ is the selected predictor in order to interpolate and extrapolate the head and static ring data shown in Figure 15a,b. For the probe head as well as for the probe static ring, a linear regression study is performed for each of the Standard Atmosphere cases based on the translated data. All these regression models together form the global predictive model. The model returns the completed anti-icing evaluation graphs which are presented in Figure 16a,b.

These graphs include the translated test data together with the predicted temperatures by the regression model. The predictions are displayed as contour plots, where the isotherms are drawn as black curves with identifying temperature labels in ${}^{{}^{\circ}}$C. The colored areas represent qualitative degrees of icing risk. Risk is considered to be high when the predicted temperature is below 0 ${}^{{}^{\circ}}$C and moderate when it is below +20 ${}^{{}^{\circ}}$C. The data are extrapolated to the left and right of the given data points to enable creation of the full contour line plot. However, this increase in uncertainty does not create an issue as the majority of regions to the right of the points lie in temperature ranges below 20 ${}^{{}^{\circ}}$C (moderate risk) and those to the left of the points are quite safe ranges of operation.

## 5. Discussion and Outlook

The heat transfer problem of a heated probe under axial flow was analytically and experimentally investigated. The analytical model is based on fundamental heat convection theory and uses the combination of two Nusselt number $\bar{Nu}$ determination models in order to estimate an average value on the probe surface. Furthermore, the system performance is investigated in Wind Tunnel B of the Chair of Aerodynamics and Fluidmechanics of the Technical University of Munich by performing temperature measurements on the probe surface for six different configuration sets of input parameters. The measurements are represented as temperature profiles along the probe surface. The measured temperatures are compared to the predictions made by the analytical model. The comparison with the analytical model shows relative errors δT between −4.4% to +3.0% for the different test configurations. Lastly, the capability of the probe heating system for maintaining the probe’s protection from icing is evaluated. This is done by predicting the temperature at the areas of the probe where the pressure ports are located—the probe head and the static ring. The test data are expanded to a series of elevation cases that consider air properties according to Standard Atmosphere data. For each case, linear regression is used to estimate the expressions that relate the probe head and static ring temperatures with the analytical model output. The resulting anti-icing evaluation graphs are used to determine the probe icing risk when operating at certain elevations and speeds.

A future investigation step should include the repetition of the presented tests under icing conditions in an icing wind tunnel. By directly studying the performance of the system in icing conditions it would be possible to check the validity of the test results translation performed during the analysis presented in this paper.

## Figures and Tables

**Table 1:** System input parameter values for the designed test configurations.

Configuration	*V* [m/s]	*q* [W]
1	25 ($V_{I}$)	10 ($q_{I}$)
2	25 ($V_{I}$)	20 ($q_{II}$)
3	25 ($V_{I}$)	30 ($q_{III}$)
4	25 ($V_{I}$)	40 ($q_{IV}$)
4	25 ($V_{I}$)	40 ($q_{IV}$)
5	35 ($V_{II}$)	40 ($q_{IV}$)
6	45 ($V_{III}$)	40 ($q_{IV}$)

**Table 2:** Distances from the probe tip to the temperature measurement points.

	*T*_1_	*T*_2_	*T*_3_	*T*_4_	*T*_5_	*T*_6_
**Distance from probe tip [mm]**	0.5	6.5	22.9	42.8	66.0	72.5

**Table 3:** Temperature measurement results from the wind tunnel experiments in [${}^{{}^{\circ}}$C].

Config.	*T*_*∞*_	*T*_1_	*T*_2_	*T*_3_	*T*_4_	*T*_5_	*T*_6_
1	22.7	36.5	46.5	88.6	58.4	37.9	34.8
2	23.1	51.1	71.6	145.3	92.1	52.2	46.2
3	23.4	66.8	97.6	200.5	124.1	65.5	57.0
4	23.8	81.3	113.5	250.7	150.9	77.0	66.3
5	24.9	59.1	77.8	219.8	123.1	62.4	55.8
6	26.3	49.7	62.7	196.8	106.8	56.4	51.2

**Table 4:** Analytical model and test results comparison.

Config.	*T*_*analytical*_ [${}^{{}^{\circ}}$C]	*T*_*test*_ [${}^{{}^{\circ}}$C]	ΔT [${}^{{}^{\circ}}$C]	δT [%]
1	61.0	64.5	−3.5	−4.4%
2	99.6	103.0	−3.4	−2.9%
3	140.1	140.7	−0.6	−0.4%
4	176.3	171.7	4.6	2.5%
5	144.6	140.2	4.4	2.8%
6	126.2	122.1	4.1	3.0%

**Table 5:** Anti-icing evaluation initial values for the probe head and the static ring containing test data from configurations 4, 5 and 6 in [${}^{{}^{\circ}}$C].

Config.	*T*_1_	*T*_5_
	Head	Static Ring
4	81.3	77.0
5	59.1	62.4
6	49.7	56.4

**Figure A1:**
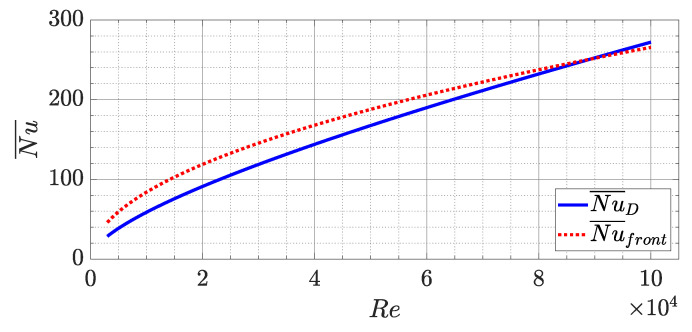
Average Nusselt number estimations for the whole sphere and the front hemisphere in the range $3.0\times 10^{3}<Re_{D}<1.0\times 10^{5}$.

## Abbreviations

The following abbreviations are used in this manuscript:

AM	Additive Manufacturing
IPS	Icing Protection System
PBF	Powder Bed Fusion
UAV	Unmanned Aerial Vehicles

## Appendix A. Additional Information of the Front Hemisphere Heat Convection

This Appendix should clarify the assumption made in Section 2.2.1 describing the actual convection of the front hemisphere, i.e. considering the first term in Equation (8) being not appropriate to fully describe the behavior on the front hemisphere.

Consequently, two ways of explanation are presented: (a) a discussion of the results in the following experimental paper *A wind tunnel investigation on the local heat transfer from a sphere, including the influence of turbulence and roughness* by Aufdermaur and Joss [16] and (b) a mathematical explanation.

### Appendix A.1. Experimental Explanation

In the mentioned journal paper [16], the authors represent in their second figure the heat transfer over a sphere for two different inflow velocities / Reynolds numbers. There, it can be seen that for the lower Reynolds number the heat transfer predominantly takes place through the front part of the sphere. In the higher Reynolds number case, the contribution of the back hemisphere to the overall heat transfer is almost similar to the front hemisphere contribution. Aufdermaur and Joss describe this as follows [16]:

*The heat transfer from this part of the sphere is dominating Re∼4000 but, as the contribution from the rear part increases faster with increasing Reynolds number, both become equally important at Re∼ 70,000.*

Coming back to the empirically derived Equation (8), a formulation for the behavior of the entire sphere is given. The two terms represent the two trends for the change in Reynolds number: The front hemisphere follows the $Re^{0.50}$ trend, and the back part the $Re^{0.92}$ trend. Hence, considering just the first term in Equation (8) is equivalent to substituting the back half term by zero. By doing so, we do not have half a sphere, but an entire sphere that is not convecting any heat to the environment through its back half. In order to actually get the appropriate value for the average Nusselt number for the front half, it is necessary to consider a sphere with two front halves. Therefore, to adequately consider the front half, we should take the same $0.42\cdot Re^{0.50}$ expression for an imaginary back hemisphere.

According to the information presented so far, an assumed expression for the average Nusselt number of a front hemisphere should not only provide a higher value than the average value for the whole sphere for low Reynolds numbers, but also should return a similar value for Re∼70,000. In Figure A1, the expressions presented in Equations (8) and (9) are plotted in order to prove the completion of these conditions after the performed assumption.

The represented data validates the assumed expression for ${\bar{Nu}}_{front}$ given that the average Nusselt numbers have the same value at Re= 90,000, which agrees well with the expected Reynolds number Re∼ 70,000.

### Appendix A.2. Mathematical Explanation

In the mathematical explanation, the expression for the convected heat through a front hemisphere is obtained from the formula originally created for a whole sphere. The experimentally found equation by Eastop and Smith [17] is introduced here again:

(A1) $${\bar{Nu}}_{D}=0.42\;Re_{D}^{0.50}+0.0035\;Re_{D}^{0.92}\;\mathrm{for}\;3.0\times 10^{3}<Re_{D}<1.0\times 10^{5}$$

This formula estimates the average Nusselt number over an entire sphere by adding two contribution terms. The first for the front hemisphere and the second for the back hemisphere. The area of the front and back hemispheres is equal, therefore each of them represent an equal portion of a whole sphere, exactly the half. Therefore, the average Nusselt number of an entire sphere could also be expressed as the arithmetic mean value between the front and back Nusselt numbers:

(A2) $${\bar{Nu}}_{D}=\frac{{\bar{Nu}}_{front}+{\bar{Nu}}_{back}}{2}$$

If the formula by Eastop and Smith is adapted to have the same form as this last formula, it is possible to define a value for the average Nusselt number separately for the front and back halves of the sphere:

(A3) $$\begin{array}{cc} {\bar{Nu}}_{D} & =0.42\;Re_{D}^{0.50}+0.0035\;Re_{D}^{0.92}=\frac{2\;(0.42\;Re_{D}^{0.50}+0.0035\;Re_{D}^{0.92})}{2}\\ \; & =\frac{0.84\;Re_{D}^{0.50}+0.007\;Re_{D}^{0.92}}{2} \end{array}$$

From a comparison of Equations (A2) and (A3), the average Nusselt number for the front hemisphere is estimated:

(A4) $${\bar{Nu}}_{front}=0.84Re_{D}^{0.50}$$
